# Effect of Boyle–Davis mouth gag placement on intracranial pressure in children undergoing tonsillectomy: A prospective observational study

**DOI:** 10.1097/MD.0000000000047697

**Published:** 2026-02-20

**Authors:** Kayacan Kaya, Başak Akca, Aysun Ankay Yilbas, Gunes Celebioglu, Banu Kilicaslan

**Affiliations:** a Department of Anesthesiology and Reanimation, Hacettepe University Faculty of Medicine, Ankara, Turkey; b Department of Anesthesiology and Reanimation, Zonguldak Maternity and Children Hospital, Zonguldak, Turkey.

**Keywords:** adenotonsillectomy, intracranial pressure, mouth gag, ONSD/ETD, optic nerve sheath diameter, pediatric anesthesia

## Abstract

Pediatric adenotonsillectomy frequently requires Boyle–Davis mouth gag placement, which may transiently increase intracranial pressure (ICP). Ultrasonographic optic nerve sheath diameter (ONSD) is a validated noninvasive marker of ICP. This study aims to investigate the impact of endotracheal intubation and Boyle–Davis mouth gag placement on ONSD, eyeball transverse diameter (ETD), and ratio of optic nerve sheath diameter to eyeball transverse diameter (ONSD/ETD) ratio in children. In this prospective observational study of 120 children (American Society of Anesthesiologists I–II, 2–18 years), bilateral ONSD and ETD were measured at post‑induction (T0), post‑intubation (T1), post‑gag placement (T2), and post‑gag removal (T3). Unadjusted profiles used repeated-measures ANOVA. Adjusted analyses used linear mixed-effects models with time and random intercept for subject; end-tidal carbon dioxide (EtCO_2_) and mean arterial pressure (MAP) were entered as time-varying covariates. Means (±standard deviation) for ONSD were 5.74 ± 0.46 (T0), 5.79 ± 0.46 (T1), 5.86 ± 0.44 (T2), 5.77 ± 0.48 (T3); for ONSD/ETD: 0.251 ± 0.021, 0.252 ± 0.020, 0.255 ± 0.020, 0.249 ± 0.021. The overall time effect was significant for both outcomes (*P* < .01). In adjusted models, T2 versus T0 remained higher for ONSD (+0.108 mm; 95% confidence interval (CI): 0.046–0.170; *P* < .001) and ONSD/ETD (+0.0033; 95% CI: 0.0003–0.0063; *P* = .03), whereas MAP was not significant and EtCO_2_ showed a modest positive association with both outcomes (ONSD: +0.007 mm/mm Hg; 95% CI: 0.002–0.012; *P* < .01). Heart rate and MAP varied over time (both *P* < .001), EtCO_2_ rose early and declined by T3 (*P* < .001). No neurologic events occurred. Mouth-gag suspension produces a small, transient rise in ONSD and ONSD/ETD that peaks at T2 and recedes after removal. Effects persisted after adjustment for EtCO_2_ and MAP, primarily suggesting contributions from positioning and suspension rather than hypercapnia. Findings support prudent positioning and monitoring in at‑risk children.

## 1. Introduction

Adenotonsillectomy is one of the most common pediatric procedures worldwide.^[1]^ Airway management typically involves endotracheal intubation and insertion of the Boyle–Davis mouth gag to optimize surgical exposure.^[2]^ While indispensable, both maneuvers can provoke hemodynamic and neurologic responses. Intubation is known to elevate mean arterial pressure (MAP) and ICP through sympathetic stimulation.^[3,4]^ The mechanical stretch and airway pressure associated with the mouth gag may produce similar effects.

Elevated ICP can compromise cerebral perfusion and, in severe cases, lead to ischemia or herniation.^[5]^ Direct ICP monitoring remains the gold standard but is invasive, resource-intensive, and impractical in routine pediatric surgery. Ultrasonographic measurement of optic nerve sheath diameter (ONSD) has emerged as a noninvasive method for measuring ICP.^[6–8]^ The optic nerve is surrounded by a dural sheath that is continuous with the intracranial subarachnoid space. As intracranial pressure increases, cerebrospinal fluid pressure can be transmitted along this compartment, resulting in distension of the optic nerve sheath that can be detected by ultrasound, most commonly measured approximately 3 mm posterior to the globe.^[9,10]^ However, cutoff values vary due to age and anthropometric differences. To address this, the ratio of optic nerve sheath diameter to eyeball transverse diameter (ONSD/ETD) has been proposed as a more robust index.^[11]^ Eyeball transverse diameter (ETD) serves as a simple surrogate of globe size. Because absolute ONSD can vary with ocular dimensions and body size, indexing ONSD to ETD may reduce inter-individual variability and improve comparability across pediatric ages and anthropometric ranges.^[11,12]^ Beyond sympathetic activation, neck extension and suspension with a mouth gag can impede jugular venous outflow, causing venous congestion and a secondary rise in ICP – mechanisms demonstrated across positioning studies and upper airway procedures.^[13–17]^ In healthy children, commonly reported ultrasonographic ONSD ranges are approximately 2.1 to 4.0 mm in infants (<1 year) and 2.4 to 4.3 mm in older children; in many pediatric studies, values above 4.0 mm (<1 year) and above 4.5 mm (>1 year) are treated as suggestive of elevated ICP.^[10,18]^ Reported ONSD/ETD values are typically clustered around 0.18 in reference cohorts, supporting its use as a size-normalized measure.^[11,12]^

Despite widespread use of the Boyle–Davis mouth gag, data on its effects on ICP in children remain limited. Recent perioperative studies using ONSD during suspension laryngoscopy further underscore the contribution of head/neck position and procedural suspension to transient ICP elevations.^[15–17]^ This study prospectively evaluated the impact of intubation and mouth gag placement on ONSD, ETD, and the ONSD/ETD ratio in pediatric patients undergoing adenotonsillectomy, with the goal of providing clinically relevant insight for anesthetic practice. This article adheres to the STROBE checklist.^[19]^

## 2. Methods

### 2.1. Study design and ethics

This prospective observational study was conducted between May 07, 2019 and October 21, 2021 at Hacettepe University. The protocol was approved by the Hacettepe University Non-Interventional Clinical Research Ethics Committee (GO 18/503). Written informed consent was obtained from parents/guardians for all participants.

### 2.2. Participants

Children < 18 years, American Society of Anesthesiologists (ASA) I to II, scheduled for adenotonsillectomy, tonsillectomy, or adenoidectomy were eligible. Exclusion criteria: prior ocular surgery, ocular/neurological disease, or clinical signs of raised ICP.

We enrolled all consecutive patients who met the eligibility criteria during the predefined period May 07, 2019 to October 21, 2021. The final sample therefore reflects complete case capture within this interval. Precision of estimates is conveyed using 95% confidence intervals (CIs).

### 2.3. Anesthesia protocol

All patients received standardized anesthesia: induction with propofol 1.5 mg/kg, rocuronium 0.6 mg/kg, fentanyl 1 to 2 μg/kg; maintenance was sevoflurane (≈0.9–1.2 age-adjusted minimum alveolar concentration) plus remifentanil infusion (titrated to hemodynamics) with pressure-controlled ventilation. We targeted end-tidal carbon dioxide (EtCO_2_) 35 to 40 mm Hg and kept MAP within 20% of pre-induction baseline. Neuromuscular blockade was reversed with sugammadex 2 to 4 mg/kg.

### 2.4. Measurements

ONSD and ETD were measured bilaterally at 4 time points: T0 (post-induction), T1 (post-intubation), T2 (after mouth-gag placement), and T3 (after mouth-gag removal). At each time point, 3 operators (in sequence) each acquired one standardized image per eye. Each operator was blinded to the others’ readings. For each eye and time point, ONSD and ETD were measured on the operator’s respective image, and the mean of the 3 operator-specific measurements was taken as the analysis value. Image acquisition at each time point required only seconds and did not interrupt anesthesia or surgery workflow. Using 3 independent acquisitions per eye reduces operator error and improves measurement reliability; averaging the 3 readings reduces random variability. Heart rate (HR), MAP, peripheral oxygen saturation (SpO_2_), and end-tidal CO_2_ (EtCO_2_) were also recorded at each timepoint.

### 2.5. Statistical analysis

Primary outcomes were ONSD and ONSD/ETD at aforementioned timepoints. The primary analysis scale was the average of the values from left and right eye.

We summarized mean ± standard deviation at each time point and tested the overall time effect using repeated-measures ANOVA with time as a within-subject factor; sphericity was assessed and Greenhouse–Geisser correction applied when violated. To evaluate potential confounding, we fit a linear mixed-effects model with time as a fixed effect and a random intercept for subject, including MAP and EtCO_2_ as time-varying covariates. We report adjusted differences for T1, T2, and T3 versus T0 and per-unit effects of MAP and EtCO_2_, *P*-values for the time contrasts were Holm-adjusted within each outcome (two-sided α = 0.05).

HR, MAP, SpO_2_, and EtCO_2_ were summarized as median [interquartile range] across T0 to T3 and compared with the Friedman test. Analyses were performed in IBM SPSS Statistics version 29.0 (IBM Corp., Armonk).

## 3. Results

### 3.1. Demographics

A total of 120 patients were included and all were complete cases: age 5.5 ± 3.2 years, weight 24.4 ± 12.3 kg, height 114.2 ± 20.5 cm; Male 64 (53.3%), Female 56 (46.7%); ASA I 105 (87.5%) and ASA II 15 (12.5%) (Table 1), Surgery duration was median 34 [interquartile range 20–48] min (mean 35.8 ± 17.5; range 10–75; n = 120).

**Table 1:** Demographic characteristics.

Variable	Value
N	120
Age (yr)	5.5 ± 3.2
Weight (kg)	24.4 ± 12.3
Height (cm)	114.2 ± 20.5
Sex	Male 64 (53.3%); Female 56 (46.7%)
ASA 1	105 (87.5%)
ASA 2	15 (12.5%)

Values are mean ± SD for continuous variables and n (%) for categorical variables.

ASA = American Society of Anesthesiologists physical status (pre-operative assessment).

### 3.2. Vital parameters

Across T0 to T3, HR and MAP changed significantly over time (both *P* < .001). Median HR rose slightly after induction and peaked at T2 (121 [108–132]), then declined to its lowest level at T3 (104 [93–114]). MAP was lowest at T1 (67 [62–72]), peaked at T2 (73 [66–79]), and remained lower again at T3 (66 [62–72]). EtCO_2_ increased early, highest at T1 (34 [31–37]), and fell by T3 (30 [27–33]) (*P* < .001). SpO_2_ was stable at 99 [99–100] throughout (*P* = .10) (Table 2).

**Table 2:** Vital parameters at each time point (T0–T3).

Parameter	T0	T1	T2	T3	*P*-value
HR	118 [103–133]	118 [102–129]	121 [108–132]	104 [93–114]	<.001
MAP	72 [66–78]	67 [62–72]	73 [66–79]	66 [62–72]	<.001
SpO_2_	99 [99–100]	99 [99–100]	99 [99–100]	99 [99–100]	.010
EtCO_2_	32 [26–36]	34 [31–37]	32 [30–35]	30 [27–33]	<.001

Values are median [IQR]. T0 = post-induction; T1 = post-intubation; T2 = after mouth-gag placement; T3 = after gag removal. *P* values are from the Friedman test using subjects with all 4 time points for each measure (two-sided α = .05).

EtCO_2_ = end-tidal carbon dioxide, HR = heart rate, IQR = interquartile range, MAP = mean arterial pressure, SpO_2_ = peripheral oxygen saturation.

### 3.3. ONSD and ETD

In the unadjusted repeated-measures analysis, Eye-averaged ONSD and ONSD/ETD means by time are shown in Table 3. Both outcomes peaked at T2 (ONSD 5.86 ± 0.44 mm; ONSD/ETD 0.255 ± 0.020) with a significant overall time effect on repeated-measures analysis of variance (both *P* < .01). Values were closer to but did not return to baseline by T3.

**Table 3:** Optic nerve sheath diameter, eyeball transverse diameter, and optic nerve sheath diameter/eyeball transverse diameter ratio.

Outcome	T0 (mean ± SD)	T1 (mean ± SD)	T2 (mean ± SD)	T3 (mean ± SD)	*P* value
ONSD (mm)	5.74 ± 0.46	5.79 ± 0.46	5.86 ± 0.44	5.77 ± 0.48	<.01
ONSD/ETD (ratio)	0.251 ± 0.021	0.252 ± 0.020	0.255 ± 0.020	0.249 ± 0.021	<.01

Values are mean ± SD. ONSD is reported in millimeters; ONSD/ETD is unitless. *P*-values test the overall time effect from repeated-measures ANOVA; Greenhouse–Geisser correction was applied when sphericity was violated (two-sided α = .05). Time points: T0 = post-induction, T1 = post-intubation, T2 = after mouth-gag placement, T3 = after gag removal.

ETD = eyeball transverse diameter, ONSD = optic nerve sheath diameter, SD = standard deviation.

After adjusting for MAP and EtCO_2_, T2 versus T0 remained higher for ONSD (+0.108 mm, 95% CI: 0.046–0.170; *P* < .001) while T1 and T3 were not significant; EtCO_2_ was positively associated with ONSD (+0.007 mm per 1 mm Hg, 95% CI: 0.002–0.012; *P* < .01), and MAP was not significant. For ONSD/ETD, T2 versus T0 also remained higher (+0.0033, 95% CI: 0.0003–0.0063; *P* = .03), with a positive EtCO_2_ association (+0.0004 per 1 mm Hg; *P* < .01). (Tables 4 and 5).

**Table 4:** Adjusted effects on optic nerve sheath diameter.

Predictor	Contrast/ Unit	Estimate (95% CI)	*P* value
Time	T1 vs T0	0.038 (−0.029 to 0.105)	.26
Time	T2 vs T0	0.108 (0.046–0.170)	<.001
Time	T3 vs T0	0.040 (−0.026 to 0.105)	.23
MAP	Per 1 mm Hg	0.001 (−0.003 to 0.004)	.71
EtCO_2_	Per 1 mm Hg	0.007 (0.002–0.012)	<.01

Linear mixed-effects model with time (categorical; T0–T3), EtCO_2_ and MAP as time-varying covariates; random intercept for subject. Reference: T0. Estimates are adjusted mean differences (mm) with 95% CI. P values for time contrasts (T1/T2/T3 vs T0) are Holm-adjusted within the outcome; covariate P values are unadjusted (two-sided α = .05). Time points: T0 = post-induction; T1 = post-intubation; T2 = after mouth-gag placement; T3 = after gag removal.

CI = confidence interval, EtCO_2_ = end-tidal carbon dioxide, MAP = mean arterial pressure, ONSD/ETD = ratio of optic nerve sheath diameter to eyeball transverse diameter.

**Table 5:** Adjusted effects on optic nerve sheath diameter/eyeball transverse diameter.

Predictor	Contrast/ Unit	Estimate (95% CI)	*P*-value
Time	T1 vs T0	0.0004 (−0.0028 to 0.0036)	.80
Time	T2 vs T0	0.0033 (0.0003–0.0063)	.03
Time	T3 vs T0	−0.0010 (−0.0041 to 0.0022)	.54
MAP	Per 1 mm Hg	0.0001 (−0.0001 to 0.0002)	.49
EtCO_2_	Per 1 mm Hg	0.0004 (0.0001–0.0006)	<.01

Linear mixed-effects model with time (categorical; T0–T3), EtCO_2_ and MAP as time-varying covariates; random intercept for subject. Reference: T0. Estimates are adjusted mean differences (unitless) with 95% CI. *P* values for time contrasts (T1/T2/T3 vs T0) are Holm-adjusted within the outcome; covariate *P* values are unadjusted (two-sided α = .05). Time points: T0 = post-induction; T1 = post-intubation; T2 = after mouth-gag placement; T3 = after gag removal.

CI = confidence interval, EtCO_2_ = end-tidal carbon dioxide, MAP = mean arterial pressure, ONSD/ETD = ratio of optic nerve sheath diameter to eyeball transverse diameter.

## 4. Discussion

Boyle–Davis mouth-gag placement was associated with small, transient increases in intracranial pressure surrogates ONSD and ONSD/ETD rose most clearly at T2 and declined after gag removal (T3). In unadjusted repeated-measures ANOVA, the overall effect of time was significant for both outcomes. In mixed-effects models that included time with EtCO_2_ and MAP as time-varying covariates, the T2 versus T0 contrast remained higher for both ONSD and ONSD/ETD, while MAP was not significant.

The peak during suspension with partial reversal after removal points to a positioning related effect. Neck extension and suspension can impede jugular venous outflow and transiently increase intracranial venous volume, producing a short-lived ONSD increase that diminishes once the load is removed.^[13–17]^ A modest positive association with EtCO_2_ is compatible with CO_2_ mediated cerebrovascular dilation, but given the small effect size and the adjusted results, this should be viewed as supportive. Sympathetic activation also occurs during both intubation and suspension^[3,4]^; the concurrent T2 rises in HR and MAP fit that interpretation, yet the absence of an independent MAP-ONSD association in adjusted models favors a primarily mechanical/positional mechanism.^[15–17]^ Minor left–right differences are physiologically plausible (jugular dominance, slight head rotation/tilt) and may also reflect technical factors (eyelid tension, probe pressure). In our study, eye-averaging mitigates the influence of such variability.

Prior adenotonsillectomy and suspension-laryngoscopy series describe reversible ONSD increases around airway manipulation with a return toward baseline after mechanical factors are relieved.^[15–17]^ Absolute ONSD thresholds vary with age and anthropometry; ratios such as ONSD/ETD provide a size-normalized index with improved comparability across children.^[6–8,11]^ Our results align with that literature but extend it by taking measurements on 4 peri-operative timepoints (post-induction, post-intubation, post-gag placement, early post-removal), using a prespecified eye-averaged primary estimand to mitigate lateral noise, and incorporating time-varying EtCO_2_ and MAP in adjusted mixed-effects models and showing that the T2 peak persists after adjustment.

In otherwise healthy ASA I to II children, the observed changes were modest, short-lived, and not associated with neurologic events. Even so, children with reduced intracranial compliance or impaired venous outflow may be more vulnerable. Practical precautions should include avoiding excessive neck extension/rotation, using the lowest effective gag tension with periodic reassessment, modest head elevation where feasible, and maintaining normocapnia. When ONSD ultrasound is available, a trend based interpretation relative to a child’s own baseline is preferable to relying on a single absolute cutoff value.^[6–8,11]^

This single center observational study included only ASA I to II children and findings may not be generalized to higher-risk populations. ONSD and ONSD/ETD are surrogates rather than direct measures of intracranial pressure, which may limit the precision of ICP assessment. Also operator dependence is an inherent limitation of ultrasound; even though we used 3 measurements by different operator per eye to reduce error. This practice aligns with reliability recommendations in the ONSD literature.^[20,21]^ Residual confounding is possible from factors not routinely recorded, such as exact gag tension, angle, and subtle head/neck position. Finally, the workflow reflects a single institutional anesthetic approach, which may limit external applicability.

Future work should quantify positioning and gag mechanics – tension, angle, and duration – to separate mechanical from other contributors; assess higher-risk pediatric subgroups with impaired autoregulation; and evaluate individualized baselines and serial ONSD/ETD trajectories as practical decision aids.

## 5. Conclusion

Mouth-gag suspension produces a transient, rise in ONSD and ONSD/ETD that attenuates after removal and remains evident after accounting for EtCO_2_ and MAP. While clinically inconsequential in healthy children, it may be significant in vulnerable populations.

## Acknowledgments

The authors acknowledge statistical consultation from Salih Ergöçen, MSc (CONSUS Biyoistatistik, Ankara).

## Author Contributions

**Conceptualization:** Başak Akca, Gunes Celebioglu.

**Data curation:** Kayacan Kaya, Aysun Ankay Yilbas.

**Formal analysis:** Başak Akca.

**Investigation:** Kayacan Kaya, Aysun Ankay Yilbas, Banu Kilicaslan.

**Methodology:** Başak Akca, Gunes Celebioglu.

**Project administration:** Başak Akca, Banu Kilicaslan.

**Resources:** Aysun Ankay Yilbas, Banu Kilicaslan.

**Supervision:** Başak Akca, Banu Kilicaslan.

**Validation:** Başak Akca, Gunes Celebioglu.

**Visualization:** Başak Akca.

**Writing – original draft:** Kayacan Kaya, Başak Akca.

**Writing – review & editing:** Kayacan Kaya, Başak Akca, Aysun Ankay Yilbas, Gunes Celebioglu, Banu Kilicaslan.
